# An Atypical Presentation of Nodular Hidradenoma of the Thigh Treated With Mohs Micrographic Surgery

**DOI:** 10.7759/cureus.85480

**Published:** 2025-06-06

**Authors:** Nabor S Mireles, Natalie H Garcia, Isabella Camacho-Hubbard, Abdul Hafeez Diwan, Jennifer S Ranario

**Affiliations:** 1 School of Medicine, Baylor College of Medicine, Houston, USA; 2 Dermatology, Baylor College of Medicine, Houston, USA; 3 Pathology and Immunology, Baylor College of Medicine, Houston, USA

**Keywords:** acrospiroma, adnexal neoplasm, adnexal tumor, hidradenoma, mohs micrographic surgery, nodular hidradenoma

## Abstract

Nodular hidradenoma (NH) is a benign adnexal tumor demonstrating both eccrine and apocrine differentiation. NH generally presents as a slow-growing, solitary, firm nodule, most commonly measuring 0.5-2 cm in diameter. It most frequently occurs on the scalp, thorax, abdomen, and gluteal regions, with a predilection for adult women in their fourth to eighth decades of life. We report the case of a 41-year-old immunocompetent female who presented with a red, 3.6 × 2.5 cm nodule on the left thigh. Due to the cystic nature of the lesion, it was initially presumed to be a benign cyst. However, as the lesion continued to grow slowly, a biopsy was performed, confirming the diagnosis of NH. Although malignant transformation is rare, it has been shown to follow an aggressive course with widely disseminated disease. Additionally, in some cases, it can be difficult to differentiate between NH and hidradenocarcinoma (HAC). Therefore, Mohs micrographic surgery (MMS) was performed to successfully remove the lesion with complete margin assessment. Currently, wide local excision (WLE) is the most common form of treatment. However, given the potential for malignancy and recurrence, we propose MMS as an alternative definitive treatment for NH, especially for tumors located in areas that would benefit from a skin-sparing surgical technique. This is among the few reported cases of NH affecting the thigh, highlighting the diagnostic challenges and the importance of considering NH in the differential diagnosis of atypical cutaneous nodules.

## Introduction

Nodular hidradenoma (NH) is a rare, benign cutaneous adnexal tumor originating from sweat glands. Although it was initially thought to arise from eccrine glands, NH has recently been shown to demonstrate apocrine differentiation [1]. Clinically, cases of NH present as slow-growing, mobile, solid or cystic nodules that measure 0.5-2.0 cm in diameter, though they have been reported to reach sizes of 6 cm or more [2]. Commonly distributed over the scalp, thorax, abdomen, and gluteal regions, NH typically affects women twice as often as men, usually in their fourth to eighth decades of life [3,4]. NHs have only rarely been reported on the extremities. Here, we present a case of NH arising on the thigh that was successfully treated with Mohs micrographic surgery (MMS).

## Case presentation

**Figure 1:**
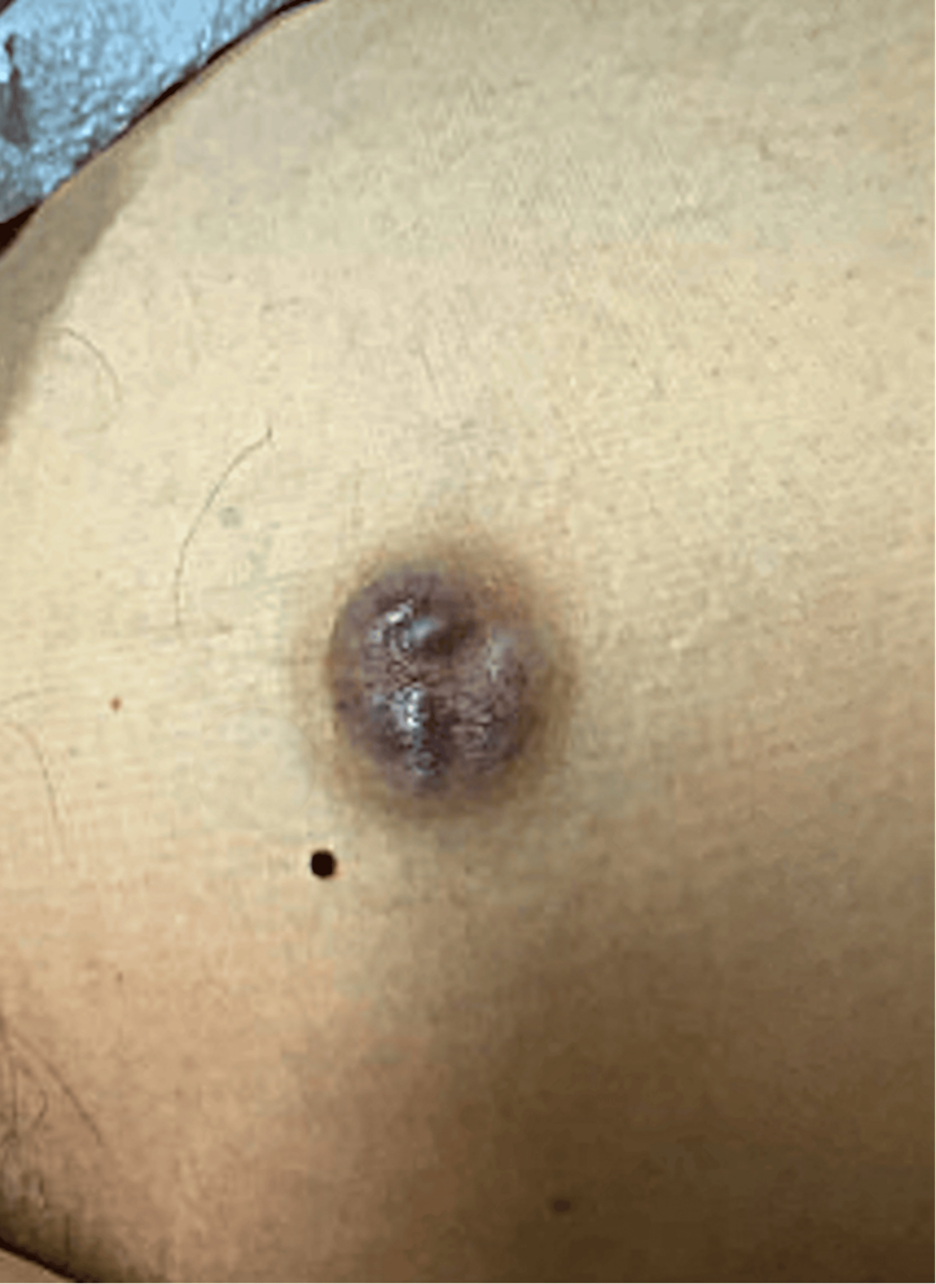
Lesion appearance prior to biopsy: violaceous nodule on the left anterior medial thigh.

A 41-year-old immunocompetent, otherwise healthy Caucasian female presented to the clinic for surgical management of a one-year history of a slowly enlarging nodule over her left anterior medial thigh. She denied any history of immunosuppression or radiation therapy. Prior to presentation, her primary care provider suspected the lesion was a benign cyst and recommended warm compresses. As the lesion persisted, she was referred to dermatology, where initial management included close monitoring. Later, the lesion grew and developed a purple hue, and she completed a course of antibiotics for what was believed to be an inflamed cyst. The patient could not recall the name of the antibiotic. At that point, she reported no pain or drainage and had no systemic symptoms. However, the lesion failed to improve with antibiotics (Figure 1), and a punch biopsy was performed, yielding a specimen measuring 0.7 × 0.2 × 0.2 cm. The differential diagnosis at the time of biopsy included an inflamed cyst and dermatofibrosarcoma protuberans. Pathology results from the biopsy confirmed a diagnosis of NH. The patient was then referred to our clinic for surgical management.

**Figure 2:**
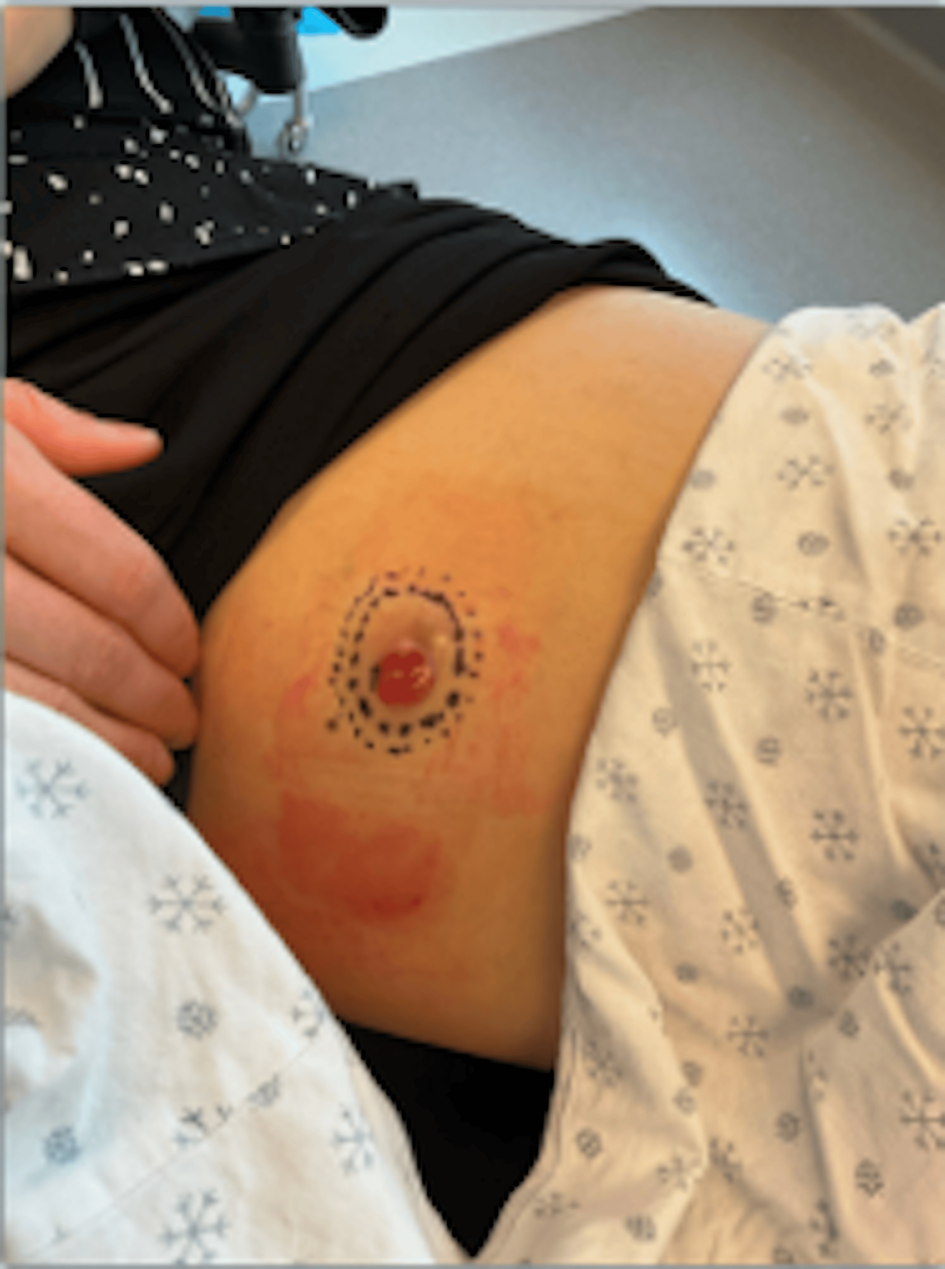
A 3.6 × 2.5 cm firm, mobile subcutaneous nodule with an overlying bright red papule following confirmatory biopsy of nodular hidradenoma on the left anterior medial thigh.

**Figure 3:**
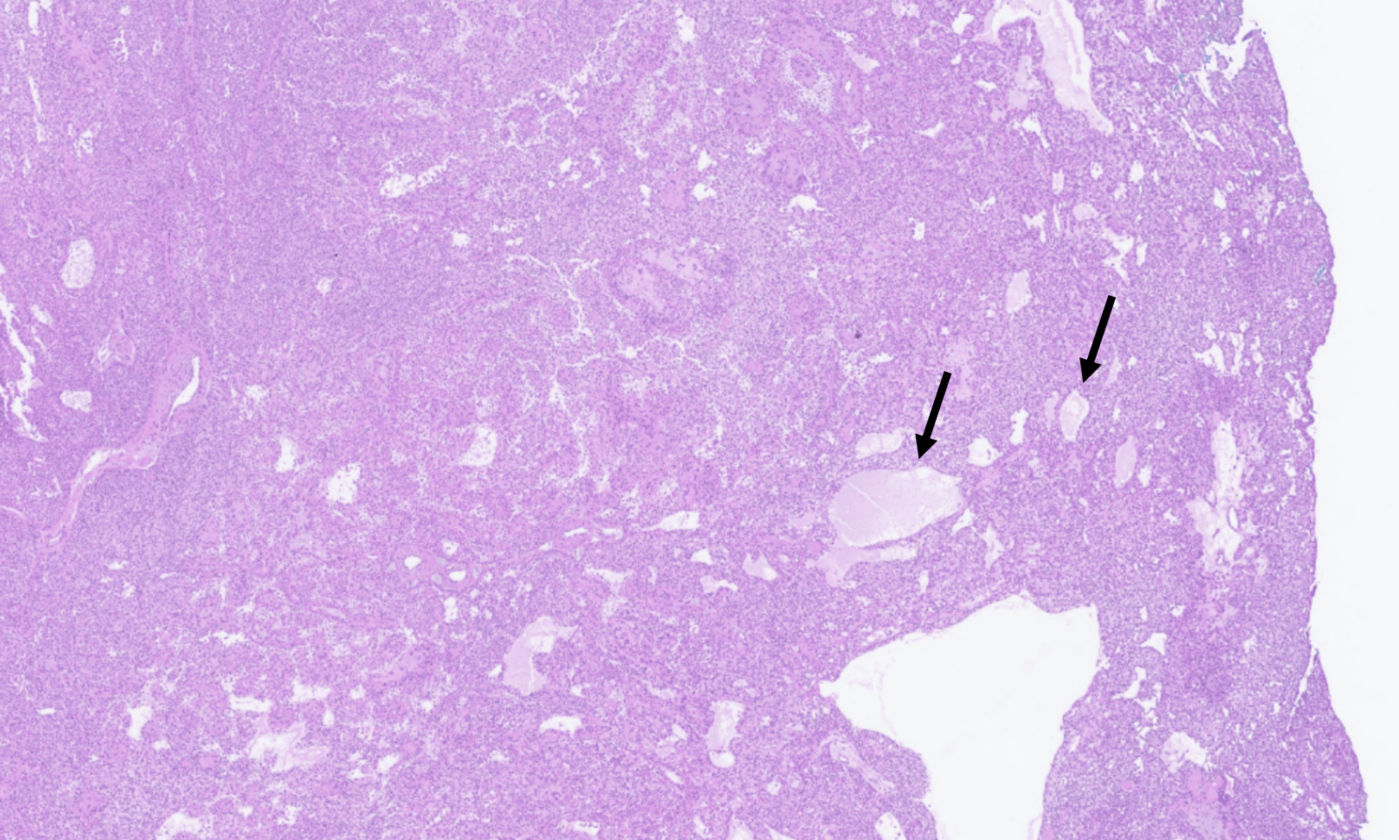
Permanent section, H&E stain (16×), showing nodular hidradenoma with poroid cells, clear cell changes, and ducts (black arrows).

**Figure 4:**
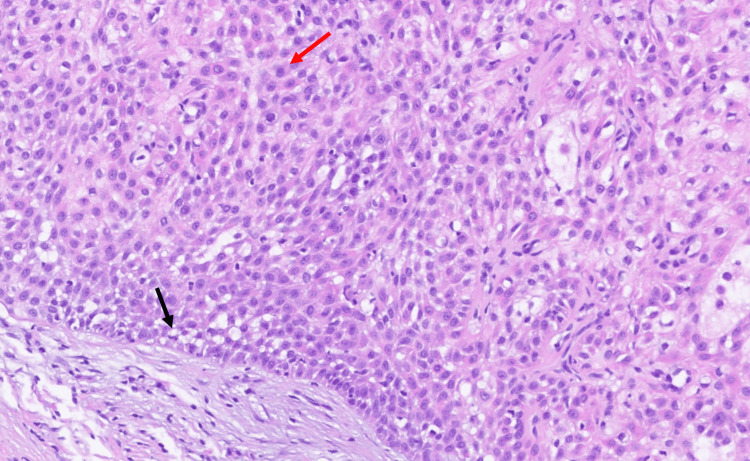
Permanent section, H&E stain (160×), showing nodular hidradenoma with poroid cells (red arrow), clear cell changes (black arrow), and ducts.

**Figure 5:**
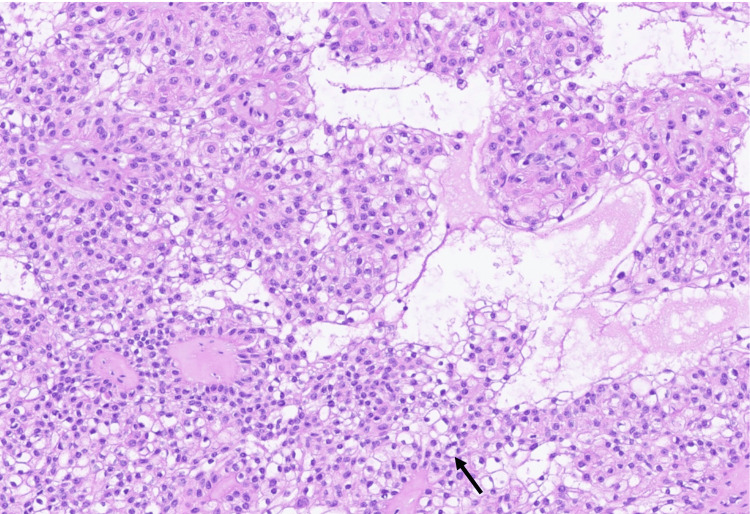
Permanent section, H&E stain (130×), showing nodular hidradenoma with clear cell change (black arrow).

The patient reported drainage from the site since the time of biopsy. Clinical examination demonstrated a firm, mobile subcutaneous nodule with an overlying bright red papule measuring 3.6 × 2.5 cm in size (Figure 2). No left inguinal lymphadenopathy was appreciated. The decision was made to proceed with MMS for complete margin evaluation. Clear margins were successfully achieved with MMS in one stage. The resultant surgical defect measured 6 × 3.6 cm, and a linear repair was performed using 4-0 poliglecaprone 25 and 4-0 polypropylene sutures. The excised specimen was then thawed and placed in formalin for permanent processing to further evaluate the tissue. The diagnosis of nodular hidradenoma was confirmed histologically. Permanent sections revealed an adnexal neoplasm composed of poroid cells with clear cell change and ductal structures (Figures 3-5), with no evidence of malignancy. The patient was advised to continue regular skin checks with her general dermatologist to monitor for recurrence.

## Discussion

NH is a benign adnexal tumor arising from sweat glands. Due to variability in its histomorphological patterns, several names have been used to describe this neoplasm: clear-cell hidradenoma, solid-cystic hidradenoma, clear-cell acrospiroma, poroid hidradenoma, and eccrine acrospiroma [5,6]. NH typically affects adults aged 40-80 years, most commonly in the seventh decade of life, though no exact incidence rate has been established [4]. It is known to predominantly affect the scalp, thorax, abdomen, and gluteal regions. Development on the lower extremities, as seen in our patient, is rare [5,7]. This unusual site highlights the diagnostic challenges posed by NH’s variable clinical presentations.

Clinically, NH presents as a slowly growing, red-, blue-, or brown-hued solitary tumor, typically measuring between 0.5-2.0 cm in size [2,7]. While rare in adults, pediatric cases are exceptionally uncommon [7]. Some lesions may produce serous discharge or ulcerate, though this is less frequent [3]. Due to its asymptomatic and slow-growing nature, diagnosis can be delayed for years [5]. Additionally, the clinical presentation of NH is nonspecific and variable, making accurate diagnosis challenging among differentials such as other adnexal tumors, vascular malformations, skin cancers, including basal cell carcinoma or melanoma, cutaneous lymphoma, cutaneous metastases, sarcomas, and epidermoid cysts, as was initially suspected in our patient [1,2,8]. Therefore, histology remains the gold standard for diagnosis of NH.

Histologically, NH is characterized as a well-circumscribed, lobulated mass primarily located in the dermis [4]. It exhibits a combination of solid and cystic components, with the solid areas composed of polyhedral cells with basophilic cytoplasm and round cells with clear, glycogen-rich cytoplasm [2]. Clear-cell hidradenoma of apocrine differentiation is the most common variant, consisting predominantly of clear or pale cells with distinct borders. Alternatively, poroid hidradenomas of eccrine differentiation are derived from small, cuboidal poroid cells and cuticular cells [9].

While NHs are known to be indolent in nature, rapid growth can be an indicator of trauma, spontaneous hemorrhage, or, most importantly, malignant transformation, which is reported in up to 7% of cases [5]. Precise incidence rates of malignant transformation remain unclear, with most cases arising de novo [4,5,7]. Among reported cases, malignant transformation has most frequently occurred on the hand, with common metastatic sites including regional lymph nodes, bones, skin, and lungs [10]. Differentiating between NH and HAC can be challenging. However, features such as an infiltrative growth pattern, deep extension, necrosis, nuclear pleomorphism, and angiolymphatic invasion are specific for malignancy [2,11]. Although PHH3 (>0.7%) and Ki-67 (>11%) have been associated with malignant transformation, immunohistochemical analysis is not routinely required, as most cases can be diagnosed using H&E staining [4]. Rare cases have been reported in which lesions initially diagnosed as histologically benign were later reclassified as malignant upon recurrence or metastasis [12]. This may occur when malignant features are present in only a small fraction of the specimen, leading to an initial misclassification. Additionally, there are variants of HAC that exhibit no cytologic atypia; in such cases, diagnosis depends on architectural features such as asymmetry, loss of circumscription, or invasion into surrounding tissue. In some instances, the only sign of malignancy may be peripheral infiltration into adjacent tissues [12].

Currently, there is no established standard of treatment for NH, though wide local excision (WLE) is typically the preferred method to decrease recurrence [4,13]. However, local recurrence after WLE is estimated at approximately 10%, likely due to incomplete excision [2]. While removing the tumor with wide margins may help reduce this risk, there is no consensus in the literature regarding optimal margin size [4]. MMS is an alternative approach that has shown promising results, with at least five case reports demonstrating no recurrence over follow-up periods ranging from 4 to 24 months [12,14,15]. MMS provides the distinct advantage of histologically examining the entire surgical margin, whereas standard excision typically evaluates less than 1-2% of the margin [16]. MMS also offers the benefits of tissue preservation and improved cosmetic outcomes, particularly in functionally or cosmetically sensitive areas such as the head and neck, where NH often occurs [4,17,18]. This approach is recommended for maximizing tumor control and improving patient outcomes, especially in cases of HAC [12,18]. A study from the Mayo Clinic involving 10 patients found that an average of 1.5 stages of MMS was sufficient to achieve clear margins for HAC, with no recurrence or metastasis observed over a 7-year follow-up period [19]. This contrasts with recurrence rates ranging from 10% to 50% reported in multiple case series of HAC treated with WLE using at least 3 cm margins [13]. While long-term studies on NH treated with MMS are limited, MMS may be an excellent treatment option for benign hidradenomas to reduce recurrence rates and the risk of malignant transformation. It may also address rare cases of HAC that are misdiagnosed as NH. Although access to MMS may be limited in resource-constrained settings, it should be considered when available [4]. We recommended MMS for our patient as the optimal treatment option given the lesion’s size, malignant potential, and risk of recurrence.

## Conclusions

NH is a rare adnexal tumor that most commonly affects the scalp, thorax, abdomen, and gluteal regions. Presentation on the thigh, as seen in this case, is atypical. This report highlights the diagnostic challenges posed by NH’s variable and nonspecific clinical presentation, emphasizing the importance of including NH in the differential diagnosis of atypical cutaneous nodules. Currently, wide local excision is the most commonly employed treatment. However, given the potential for malignancy and recurrence, we propose MMS as an alternative definitive treatment for NH, particularly for tumors located in areas where a skin-sparing surgical technique would be beneficial.
